# Erythropoietin levels in patients with sleep apnea: a meta-analysis

**DOI:** 10.1007/s00405-017-4483-1

**Published:** 2017-03-09

**Authors:** Xiao-Bin Zhang, Yi-Ming Zeng, Hui-Qing Zeng, Hua-Ping Zhang, Hui-Ling Wang

**Affiliations:** 1 The Second Clinical College of Fujian Medical University, No. 134, Zhongshan Bei Road, Licheng District, Quanzhou City, Fujian Province 362001 P. R. China; 2 Department of Respiratory Medicine, Zhongshan Hospital, Xiamen University, Xiamen City, Fujian Province P. R. China; 3 Department of Respiratory Medicine, The Second Affiliated Hospital of Fujian Medical University, Quanzhou City, Fujian Province P. R. China

**Keywords:** Sleep apnea, Erythropietin, Meta-analysis

## Abstract

Currently available data regarding the blood levels of erythropoietin (EPO) in sleep apnea (SA) patients are contradictory. The aim of the present meta-analysis was to evaluate the EPO levels in SA patients via quantitative analysis. A systematic search of Pubmed, Embase, and Web of Science were performed. EPO levels in SA group and control group were extracted from each eligible study. Weight mean difference (WMD) or Standard mean difference (SMD) with 95% confidence interval (CI) was calculated by using fixed-effects or random effect model analysis according to the degree of heterogeneity between studies. A total of 9 studies involving 407 participants were enrolled. The results indicated that EPO levels in SA group were significantly higher than that in control group (SMD 0.61, 95% CI 0.11–1.11, *p* = 0.016). Significantly higher EPO levels were found in patients with body mass index <30 kg/m^2^, and cardiovascular complications in the subsequent subgroup analysis (both *p* < 0.05). High blood EPO levels were found in SA patients in the present meta-analysis.

## Introduction

Sleep apnea (SA), namely, is the absence of oronasal air flow during sleep. The pathophysiologic characteristics of SA are chronic intermittent hypoxia and sleep fragmentation [1]. SA is a highly prevalent disorder among adults [2]. SA is divided to obstructive sleep apnea (OSA) and central sleep apnea (CSA). The former is characterized by recurrent collapse of upper airway, whereas the latter is caused by the unstable ventilation drive [1, 3]. Abundant evidence confirmed that SA is firmly associated with increased risk of cardiovascular disease and mortality [4, 5]. Studies also suggested that SA might influence the levels of hematocrit [6] and blood viscosity [7]. The correlation between SA and polycythemia has also been reported [8].

Erythropoietin (EPO) is a glycoprotein hormone which is synthesized primarily by the kidney in adult. It is widely recognized that EPO may stimulate erythroid stem cells of the bone marrow to proliferate and differentiate. EPO has also been identified to play an important role in the mechanism of cardiovascular diseases. Robust evidence shows that EPO appears to be released in response to hypoxia [9].

Whether the EPO levels in SA patients being changed or not remains controversial. Some studies demonstrated that EPO concentrations were no different between SA patients and normal subjects [10, 11]. In other studies, however, authors claimed that EPO levels were significantly increased in SA subjects [12, 13]. Furthermore, it has been suggested that continuous positive airway pressure (CPAP) treatment might normalize the diurnal EPO levels in SA patients [14].

The primary aim of the present meta-analysis was to evaluate the EPO levels in SA patients via quantitatively analysis the present available literature.

## Materials and methods

The present meta-analysis was conducted following the guideline of the Preferred Reporting Items for Systematic reviews and Meta-Analysis (PRISMA) [15].

### Search strategy

The electronic databases, namely PubMed, Embase, and Web of Science, were searched by using the following terms: *sleep apnea* OR *sleep-disorder breathing*, and *erythropoietin* from inception to May, 4, 2016. No language restrictions were applied. References from included studies were also perused.

### Literature selection criteria

Two reviewers independently selected the available studies. Studies which met the following criteria were enrolled into the present meta-analysis: (1) participants included in the study were adults; (2) diagnosis of SA was according to polysomnography (PSG); (3) EPO levels were reported both in SA group and control group. Editorials, reviews, case reports, congress articles, and animal studies were excluded. An email to the corresponding author was written if the essential data of study was ambiguous or not presented. After two no response attempt, the study was also ruled out. The consensus was obtained through a meeting with all authors if any discrepancy were presented between the two reviewers.

### Literature quality and data extraction

Evidence level was defined in accordance with Oxford Centre for Evidence-based Medicine (CEBM)-Levels of Evidence [16, 17]. The data of the included articles were extracted by two reviewers independently. The following items were listed: first author, publication year, country, sample size, and the clinical characteristics of the participants, such as age, male percentage, body mass index (BMI), complications, PSG parameters and EPO levels in each group.

### Statistical analysis

Stata version 12.0 and Review Manager 5.2 were applied for statistical analysis. The *I* ^2^ was obtained to express the heterogeneity between studies. If *I* ^2^ > 50%, indicating that moderately or highly heterogeneous existed, a random effects model was conducted to estimate the effect size (Standard mean difference, SMD; 95% confidence interval, 95% CI); if *I* ^2^ ≤ 50%, fixed-effects model was used to obtain the weighted mean difference (WMD, 95% confidence interval, 95% CI) [18]. Furthermore, subgroup analysis was performed to assess the influence of gender, BMI, AHI, cardiovascular disease, and time of exsanguinate blood on EPO levels. Since these variables of other type of SA [19, 20] and ODI value [21, 22], were only reported in two included studies, subgroup analysis were not performed stratified by these two variables. Sensitivity analysis was also conducted to evaluate the influence of each study on overall effect size. Potential publication bias was showed with funnel plot, and tested by Begg’s test and Egger’s test [23]. Statistical significance was confirmed if *p* value <0.05.

## Results

### Literature search

**Fig. 1:**
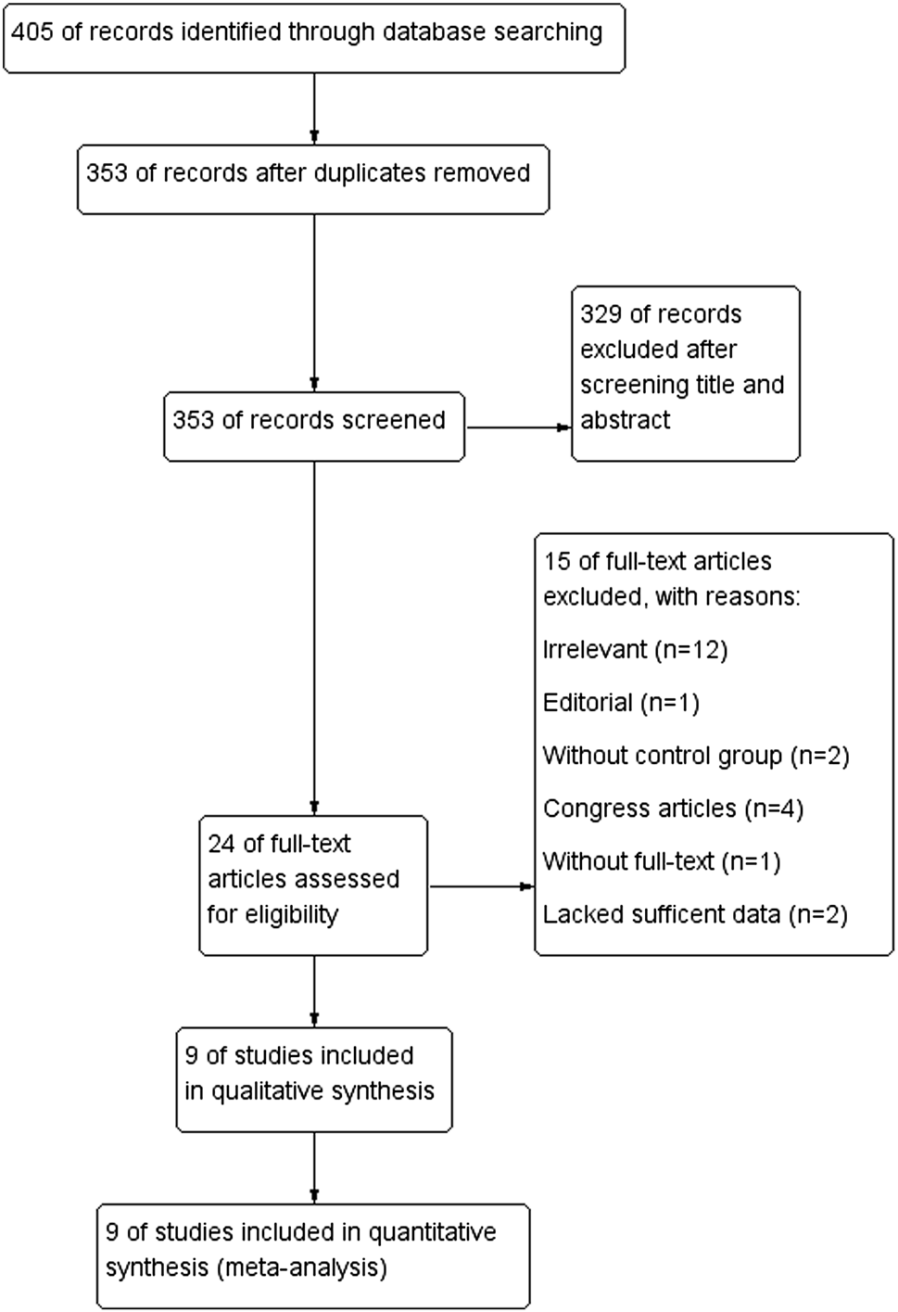
Study flow diagram

A total of 405 studies were identified by electronic search for initial scrutiny. After removing duplicated records and reviewing the titles and abstracts, 24 studies were considered worthy of further full-text scrutiny. Of the 24 studies, 15 studies were subsequently excluded (see detail in Fig. 1). Finally, 9 studies [10–13, 19–21, 24, 25] involving 407 participants were included into the present meta-analysis.

### Characteristics of included studies

Tables 1 and 2 outline the characteristics of the subjects in each study. The evidence levels were 2b (both evidence levels and recommendation grade were moderate according to CEBM) in all studies. Sample size in each study was relatively small (less than 100). The predominant type of SA was obstructive sleep apnea (OSA) in most of the included studies. Except for one study, all SA patients in the included studies suffered severe SA (the apnea hypopnea index was more than 30 events/h). The exact EPO data in one study was obtained via contacting the corresponding author [25].

**Table 1:** Characteristics of included studies

Study no.	Author	Publication year	Country	Study design	Evidence level	Sample size	
						SA group	Control group
1	McKeon	1990	Australia	Cross section	2b	23	36
2	Cahan	1992	USA	Cross section	2b	12	9
3	Pokala	1995	USA	Cross section	2b	8	8
4	Imagawa	2001	Japan	Cross section	2b	41	45
5	Wang	2004	China	Cross section	2b	18	16
6	Ryan	2005	Ireland	Cohort study	2b	19	14
7	Calvin	2010	Chile	Cross section	2b	14	15
8	Cifitci	2011	Turkey	Cohort study	2b	69	17
9	Kukwa	2013	Poland	Cross section	2b	14	23

*SA* sleep apnea

**Table 2:** Characteristics of included studies

Study no.	First author	Age (years, SA group/control group)	Male (%, SA group/control group)	BMI (kg/m^2^, SA group/control group)	AHI (events/h, SA group/control group)	ODI (events/h, SA group/control group)	Type of SA	Cardiovascular complications	Time of exsanguinate blood for EPO detection	EPO (Mean ± SD)		EPO unit
										SA group	Control group	
1	McKeon	52.8/56.0	88.4/81.0	31.9/29.2	33.8/3.4	125/0.7	OSA	No	Evening and morning	11.8 ± 9.1	10.7 ± 7.1	mU/ml
2	Cahan	47/54	100	NA	59/1.8	NA	OSA	Hypertension	Morning (8am)	46 ± 35	17 ± 8	mU/ml
3	Pokala	44.4/35.4	100.0/62.5	42.9/36.8	50.8/2.2	NA	OSA	NA	Evening (10 pm)	21.2 ± 7.4	22.8 ± 7.3	mU/ml
4	Imagawa^a^	NA	NA	NA	30–49/<5	NA	OSA	NA	NA	17 ± 20	10 ± 5	mU/ml
5	Wang^b^	48/43	100/100	NA	≥40/<5	NA	OSA	No	Morning(5am)	1.63 ± 0.26	1.47 ± 0.08	ug/l
6	Ryan^c^	39/40	100/100	32.5/31.0	48.5/1.0	46/2	OSA	No	Morning(after PSG)	13.4 ± 4.2	17.8 ± 12.5	mU/ml
7	Calvin^d^	65.7/59.9	93/53	28.3/27.2	45.0/3.6	NA	CSA	HF	Morning(post PSG)	21.8 ± 9.1	16.5 ± 6.0	mU/ml
8	Cifitci	53.27/51.5	71.0/64.7	30.9/29.1	48.4/1.89	NA	OSA	No	Morning	10.8 ± 6.0	9.7 ± 5.1	NA
9	Kukwa^e^	53.3/50.2	100/100	27.9/27.3	18.7/1.8	NA	SDB	AMI	NA	24.3 ± 7.7	10.4 ± 3.2	NA

*SA* sleep apnea, *BMI* body mass index, *AHI* apena hypopnea index, *ODI* oxygen desaturation index, *EPO* Erythropoietin, *SD* stand deviation, *PSG* polysomnography, *NA* no available, *AMI* acute myocardial infarction

^a^AHI<5 in control group, AHI30-49 in OSA group

^b^EPO data at 5:00 am were extracted

^c^Exact EPO data was obtained by contacting the corresponding author via email

^d^SDB group were all central sleep apnea patients

^e^EPO data was extracted at day 1

### Pool analysis of the difference in EPO between SA group and control group

**Fig. 2:**
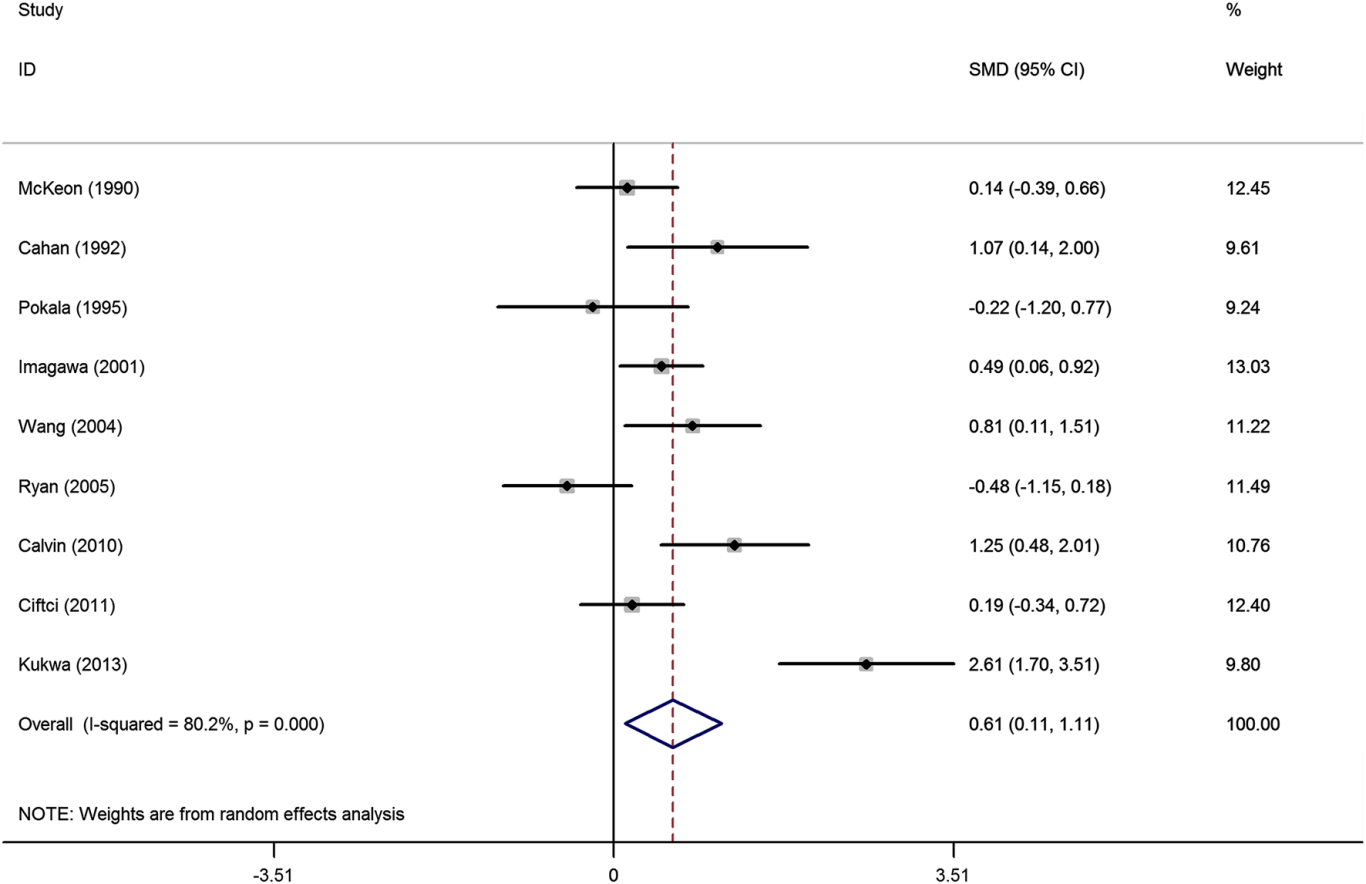
Pool analysis of the difference in EPO between SA group and control group

As the *I* ^2^ (80.2%) was significantly high, obviously heterogeneous existed between studies, thus random effect model was conducted to explore the difference in EPO between groups. Figure 2 illustrates that EPO levels in the SA group were significantly higher than that in control group (SMD 0.61, 95% CI 0.11–1.11, *p* = 0.016).

### Subgroup analysis stratified by various clinical parameters

Table 3 outlines the subgroup analysis stratified by different parameters. Results showed that there were significantly higher EPO levels in patients with BMI <30 kg/m^2^, and cardiovascular complications. EPO levels were increased in patients with SA regardless of AHI levels. Gender, and exsanguinate time had no influence on EPO in the further subgroup analysis.

**Table 3:** Results of subgroup analysis

Subgroup	No. of study	Heterogeneity		WMD	
		*I* ^2^	*p*	WMD (95% CI)	*p*
Gender					
Male	4	70.4	0.000	0.35 (−0.28–0.98)	0.279
Male and female	4	88.0	0.000	0.93 (−0.22–2.08)	0.111
BMI ≥30 kg/m^2^ in OSA group					
Yes	4	0.0	0.399	−0.01 (−0.32–0.30)	0.930
No	2	80.3	0.024	1.90 (0.57–3.24)	0.005
AHI ≥30 events/h					
Yes	8	60.9	0.012	0.38 (0.02–0.75)	0.040
No	1	–	–	2.61 (1.70–3.51)	0.000
Cardiovascular disease					
Yes	3	70.8	0.033	1.63 (0.71–2.55)	0.001
No	4	56.5	0.075	0.16 (−0.30–0.61)	0.501
Time of exsanguinate blood					
Morning	5	73.9	0.004	0.53 (−0.09–1.14)	0.092
Evening	1	–	–	−0.22 (−1.2–0.77)	0.664
Morning and evening	1	–	–	0.14 (−0.39–0.66)	0.604

*WMD* weighted mean difference, *CI* confidence interval, *BMI* body mass index, *OSA* obstructive sleep apnea, *CSA* central sleep apnea, *SDB* sleep-disorder breathing

### Sensitivity analysis

**Fig. 3:**
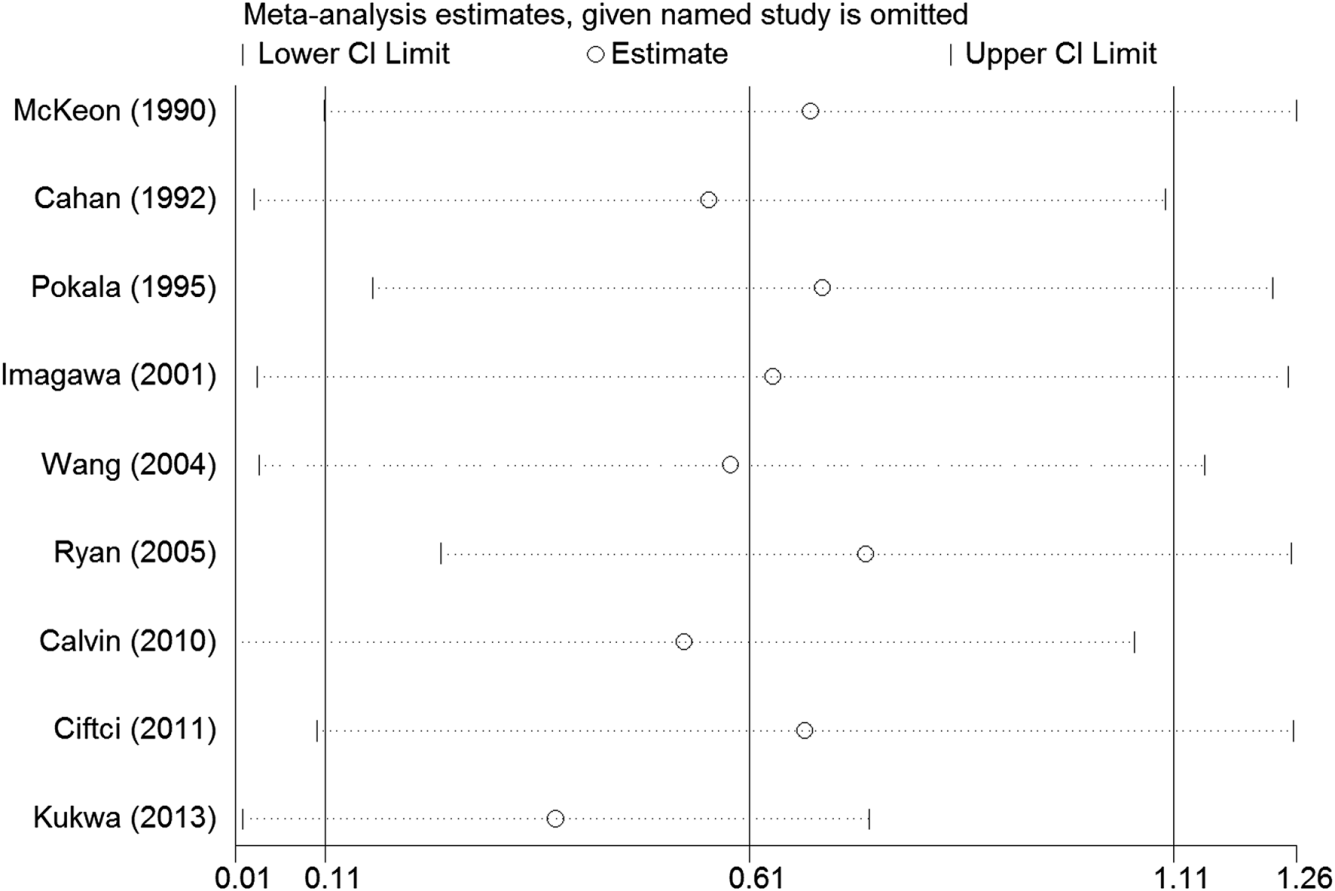
Sensitivity analysis

Sensitivity analysis showed that individual study had no influence on the overall effect size (Fig. 3).

### Publication bias

**Fig. 4:**
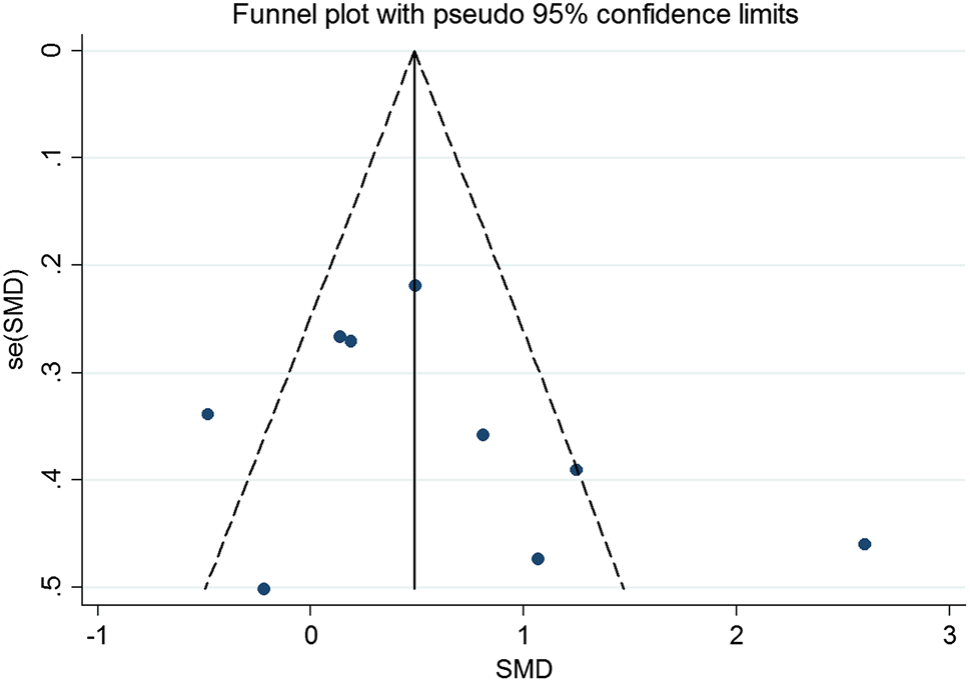
Publication bias

Figure 4 shows that publication bias seem to exist, however, both Begg and Egger tests proved that no publication bias existed in the present study (*p* = 0.293 and 0.466, respectively).

## Discussion

The present meta-analysis with 9 studies involving 407 participants demonstrated that, compared with normal subjects, EPO levels were increased in patients with SA, especially in those with low BMI, and cardiovascular complications. 

EPO, a glycoprotein hormone with 30.4 kDa relative molecular mass, is composed of 165 amino acids. It is mainly produced by the kidney and liver in adult [26]. EPO can bind to erythroid progenitor cell surface receptor, then leading to the activation of several signal pathways, such as the Ras/mitogen-activated kinase pathway, *etc* [27]. After stimulation of certain genes expression, erythropoietic progenitor cells can proliferate and differentiate to mature red blood cells. The vital function of EPO is of regulation the blood oxygen levels via adjusting the circulating erythrocytes number [26]. Diurnal variation of serum EPO levels can be detected in normal subjects: the nadir occurred at daytime, the peak concentration happened early in the morning, but this phenomenon could not be observed in chronic obstructive pulmonary disease [28, 29].

Serum levels of EPO can be influenced by various factors. The most important one is hypoxia. Evidence showed that elevated serum EPO levels are an adaptive response of human body to hypoxia. A previous study indicated that COPD patients, characterized by sustained hypoxia, had elevated serum EPO levels [30]. The pathophysiological mechanism of intermittent hypoxia is similar to ischemia/reperfusion injury. Several studies indicated that pre-exposure to intermittent hypoxia can protect the myocardial tissue against ischemia/reperfusion injury [31, 32]. Similarly to sustained hypoxia, intermittent hypoxia has also been found to play a magnificent role in the regulation of EPO levels. An experimental study illustrated that EPO was increased significantly when rats were exposed to intermittent hypoxia for 1–3 weeks [33]. The vital pathophysiological characteristic of SA is intermittent hypoxia. However, whether the EPO levels are increased or not in SA patients remained controversial. In addition, some interventional studies on the effect of CPAP treatment on EPO levels have shown contradictory results. Cahan et al. [14] showed that CPAP treatment might attenuate diurnal EPO levels in SA patients. A study by Ryan and coworkers [25] demonstrated that the EPO levels in OSA patients had no alternation after 6 weeks of CPAP treatment. Multicenter, randomized-control interventional study is needed to clarify the definitive effect of CPAP on EPO levels.

The subgroup analysis of the present study indicated that SA patients with cardiovascular disease had significantly higher EPO levels. We speculated that elevated EPO levels in patients with SA and cardiovascular disease were an adaptive response against intermittent hypoxia [31]. Previous studies claimed that EPO levels had diurnal variation [12, 24]. The present meta-analysis was inconsistent with those studies, in that we could not observe the circadian fluctuation of EPO in SA patients. We also failed to explain the phenomenon that when compared to patients with high BMI, EPO was increased in patients with low BMI. The low numbers of included studies and small sample size might partly contribute to those phenomena. Further investigation is required to clarify those aforementioned phenomena.

Several limitations of the present meta-analysis should be emphasized. First, the most significant limitation was the severe heterogeneity between included studies, showing a high variation of the results among each study. Second, the individual study is relatively low-level evidence. Third, although a highly sensitive search strategy for the potentially eligible studies was applied, some studies may still be overlooked. Fourth, the relative small sample size of each included study might restrict the generalizability of the results. Fifth, except two included studies [19, 20] (subjects in one study was CSA, the other one was sleep-disorder breathing), subjects in the most remain studies were OSA, it was difficult for us to evaluate the influence of SA type on serum EPO levels. Finally, although statistical significance was not observed, it was still hard to rule out the publication bias.

In conclusion, the present meta-analysis confirmed that elevated EPO levels were found in SA patients. We speculated that increased EPO might be associated with increased risk of subsequent cardiovascular diseases in SA patients.
